# Comprehensiveness of State Insurance Laws and Perceived Access to Pediatric Mental Health Care

**DOI:** 10.1001/jamanetworkopen.2024.26402

**Published:** 2024-08-12

**Authors:** Ashley A. Foster, Jennifer A. Hoffmann, Megan D. Douglas, Michael C. Monuteaux, Katherine E. Douglas, Teal W. Benevides, Joel D. Hudgins, Amanda M. Stewart

**Affiliations:** 1 Department of Emergency Medicine, University of California, San Francisco; 2 Division of Emergency Medicine, Ann and Robert H. Lurie Children’s Hospital of Chicago, Chicago, Illinois; 3 Department of Community Health and Preventive Medicine, National Center for Primary Care, Morehouse School of Medicine, Atlanta, Georgia; 4 Division of Emergency Medicine, Boston Children’s Hospital, Boston, Massachusetts; 5 Institute of Public and Preventive Health, Augusta University, Augusta, Georgia; 6 Division of Emergency Medicine, Children’s National Hospital, Washington, District of Columbia

## Abstract

**Importance:**

Many US children and adolescents with mental and behavioral health (MBH) conditions do not access MBH services. One contributing factor is limited insurance coverage, which is influenced by state MBH insurance parity legislation.

**Objective:**

To investigate the association of patient-level factors and the comprehensiveness of state MBH insurance legislation with perceived poor access to MBH care and perceived inadequate MBH insurance coverage for US children and adolescents.

**Design, Setting, and Participants:**

This retrospective cross-sectional study was conducted using responses by caregivers of children and adolescents aged 6 to 17 years with MBH conditions in the National Survey of Children’s Health and State Mental Health Insurance Laws Dataset from 2016 to 2019. Data analyses were conducted from May 2022 to January 2024.

**Exposure:**

MBH insurance legislation comprehensiveness defined by State Mental Health Insurance Laws Dataset (SMHILD) scores (range, 0-7).

**Main Outcomes and Measures:**

Perceived poor access to MBH care and perceived inadequacy of MBH insurance were assessed. Multivariable regression models adjusted for individual-level characteristics.

**Results:**

There were 29 876 caregivers of children and adolescents with MBH conditions during the study period representing 14 292 300 youths nationally (7 816 727 aged 12-17 years [54.7%]; 8 455 171 male [59.2%]; 292 543 Asian [2.0%], 2 076 442 Black [14.5%], and 9 942 088 White [69.6%%]; 3 202 525 Hispanic [22.4%]). A total of 3193 caregivers representing 1 770 492 children and adolescents (12.4%) perceived poor access to MBH care, and 3517 caregivers representing 1 643 260 of 13 175 295 children and adolescents (12.5%) perceived inadequate MBH insurance coverage. In multivariable models, there were higher odds of perceived poor access to MBH care among caregivers of Black (adjusted odds ratio [aOR], 1.35; 95% CI, 1.04-1.75) and Asian (aOR, 1.69; 95% CI, 1.01-2.84) compared with White children and adolescents. As exposures to adverse childhood experiences (ACEs) increased, the odds of perceived poor access to MBH care increased (aORs ranged from 1.68; 95%, CI 1.32-2.13 for 1 ACE to 4.28; 95% CI, 3.17-5.77 for ≥4 ACEs compared with no ACEs). Compared with living in states with the least comprehensive MBH insurance legislation (SMHILD score, 0-2), living in states with the most comprehensive legislation (SMHILD score, 5-7) was associated with lower odds of perceived poor access to MBH care (aOR, 0.79; 95% CI, 0.63-0.99), while living in states with moderately comprehensive legislation (score, 4) was associated with higher odds of perceived inadequate MBH insurance coverage (aOR, 1.23; 95% CI, 1.01-1.49).

**Conclusions and Relevance:**

In this study, living in states with the most comprehensive MBH insurance legislation was associated with lower odds of perceived poor access to MBH care among caregivers for children and adolescents with MBH conditions. This finding suggests that advocacy for comprehensive mental health parity legislation may promote improved child and adolescent access to MBH services.

## Key Points

**Question:**

Is comprehensiveness of state insurance legislation associated with perceived access to mental and behavioral health care for children and adolescents?

**Findings:**

In this cross-sectional study of 29 876 caregivers representing 14 292 300 children and adolescents, those living in states with the most comprehensive state insurance legislation had 0.79 lower adjusted odds of reported poor access to mental and behavioral health care compared with those living in states with less comprehensive legislation.

**Meaning:**

This study found that comprehensiveness of state mental health insurance legislation was associated with perceptions of child and adolescent access to mental and behavioral health services.

## Teaser

This cross-sectional study investigates the association of the comprehensiveness of state mental health insurance laws with caregivers’ perception of access and insurance coverage for child and adolescent mental health care.

## Introduction

Mental and behavioral health (MBH) conditions are common in children and adolescents in the US.^1,2^ However, accessing MBH treatment for children and adolescents can be challenging. Between 50% and 70% of children and adolescents with a treatable MBH condition do not receive care from a mental health professional.^1^ Access to MBH care may be influenced by social determinants of health,^3^ reflecting environments in which children and adolescents are born, grow, and live, and exposure to adverse childhood experiences (ACEs).^4,5^ Additional factors that limit access to MBH services include shortages of pediatric mental health clinicians in the US^6,7^ and inadequate child and adolescent insurance coverage for MBH services.^8^

The Mental Health Parity and Addiction Equity Act of 2008 and Patient Protection and Affordable Care Act of 2010 aimed to improve insurance coverage for MBH services. These federal laws required health plans to provide mental health and substance use benefits at parity with physical health benefits so as to reduce out-of-pocket spending and increase access to MBH services.^9^ However, children and adolescents continue to experience gaps in coverage for certain MBH conditions,^10^ and enforcement of federal parity laws varies across states.^11^ State-level mental health insurance laws can potentially assist in closing gaps in federal insurance coverage requirements and strengthen enforcement of federal laws; however, laws vary in comprehensiveness by state.^10^ Prior literature highlights the association of insurance status and type of insurance with access to mental health care, and there are differences in unmet mental health needs based on insurance type.^12,13^ However, there is limited understanding of how state-level mental health insurance parity laws are associated with perceptions of coverage adequacy for children and adolescents with MBH conditions across different insurance types or whether comprehensiveness of mental health insurance laws is associated with perceived access to MBH care for children and adolescents.

The number of children and adolescents experiencing mental and behavioral health challenges has increased rapidly in the US, leading the American Academy of Pediatrics and other professional organizations to declare a national emergency in 2021.^14^ Understanding how state legislation is associated with access to MBH care for children and adolescents is a priority. Additionally, identification of individual-level factors that may be associated with perceived access to MBH care and insurance coverage is essential given that policies may be associated with different outcomes by subgroup.^15^

Thus, we aimed to determine the association between comprehensiveness of state mental health insurance legislation and perceived access to pediatric MBH care among caregivers of US children and adolescents, adjusting for individual-level factors that reflect demographics and social determinants of health. Additionally, because of previously described differences in perceived unmet health care needs by insurance type,^16^ we sought to determine the association between comprehensiveness of state mental health insurance legislation and caregiver-perceived adequacy of MBH insurance coverage among US children and adolescents with differing types of insurance coverage.

## Methods

### Study Design and Setting

We conducted a retrospective cross-sectional study using the National Survey of Children’s Health (NSCH) and State Mental Health Insurance Laws Dataset (SMHILD) from 2016 to 2019. Data analysis was conducted May 2022 to January 2024. The NSCH is a caregiver-proxy survey conducted by the US Census Bureau examining 1 child or adolescent from each randomly selected household to estimate national-level data on US child and adolescent health.^17,18^ The SMHILD describes comprehensiveness of state MBH insurance laws, enabling comparison of the state legislative landscape across US states.^10^ The SMHILD used a validated legal coding instrument consisting of 6 questions across 4 themes (parity, mandated coverage, mental health condition definition, and enforcement-compliance), producing a composite comprehensiveness score for each state that ranges from 0 (least comprehensive) to 7 (most comprehensive) (eTable 1 in Supplement 1).^10,15^ Components used to generate an SMHILD score and variation in these components across states have been previously described.^10^ This study was deemed exempt from review and participant informed consent by the Boston Children’s Hospital Institutional Review Board because the study does not represent human participant research and met regulatory requirements necessary to obtain a waiver of informed consent and authorization. The study followed the Strengthening the Reporting of Observational Studies in Epidemiology (STROBE) reporting guideline.

### Survey-Level Data

We included survey responses from caregivers of children and adolescents aged 6 to 17 years with an MBH condition.^1^ Presence of an MBH condition was defined by positive caregiver responses to survey items that the child or adolescent had 1 of the following MBH conditions: history of anxiety, attention-deficit/hyperactivity disorder, autism spectrum disorder, conduct or behavioral disorder, depression, intellectual disability and developmental delay,^19,20^ substance use disorder, Tourette syndrome, or another MBH condition (eTable 2 in Supplement 1).

Individual-level covariates were chosen based on prior literature,^5,21,22,23^ using the Andersen model of health care use as a conceptual framework to select predisposing factors, enabling factors, and need factors.^24^ Factors associated with a predisposition to using medical care included age, sex, race, and ethnicity.^1,23^ Demographic information, including race (American Indian or Alaska Native alone, Asian alone, Black or African American alone, Native Hawaiian and Other Pacific Islander alone, White alone, some other race alone, and 2 or more races) and ethnicity (not Hispanic or Latino origin and Hispanic or Latino origin), is provided by caregivers completing the NSCH questionnaire.^25^ Race and ethnicity were considered as social constructs rather than genetic or biological categories and were included to reflect the influence of structural racism on access to MBH resources.^26,27^ As contributors to access to care, child immigration status, caregiver immigration status, primary household language, number of ACEs, family structure, caregiver education, and caregiver mental health were included.^1,21,22,23,28,29,30,31^ Perceived and measured need for health care were represented by the general health of the child or adolescent and visits to a health care professional in the past 12 months.

### Outcome Measures

The primary exposure was comprehensiveness of state MBH insurance laws defined using the SMHILD score. This score was categorized as 0 to 2, 3, 4, and 5 to 7, with the lowest and highest scores collapsed so that no score category represented less than 5% of the study sample.

Outcome measures were perceived poor access to MBH care and perceived inadequacy of MBH insurance coverage. Poor access to MBH care was a composite measure defined by responses to the following NSCH items: (1) “How much of a problem was it to get the mental health treatment or counseling that this child needed” with response of “Big problem” from 2016 to 2017 and “How difficult was it to get the mental health treatment or counseling that this child needed?” with responses “Very difficult” or “It was not possible to obtain care” from 2018 to 2019 (question wording and response options varied slightly across years); (2) “During the past 12 months, was there any time when this child needed health care but it was not received?” with responses of “Yes” and “Mental health services”; and (3) “During the past 12 months, has this child received any treatment or counseling from a mental health professional?” with a response of “No, but this child needed to see a mental health professional.” Inadequate MBH insurance coverage was defined by the item “Thinking specifically about this child’s mental or behavioral health needs, how often does this child’s health insurance offer benefits or cover services that meet these needs?” with responses of “Sometimes” or “Never.”

### Statistical Analysis

We described legislation comprehensiveness scores, perceived access to MBH care, and perceived MBH insurance coverage adequacy by state. We examined differences in perceived access to MBH care and MBH insurance coverage adequacy by legislation comprehensiveness using χ^2^ tests.

We used multivariable regression models to analyze associations of SMHILD scores with perceived poor access to MBH care and perceived inadequacy of MBH insurance coverage, adjusted for individual-level variables. White children and adolescents were used as the reference group in multivariable models because they were the highest proportion (69.6%) of children and adolescents in our sample. Analyses were conducted within the context of NSCH survey design characteristics to generate population-level estimates. Because the legislation categorized by the SMHILD score mainly applies to commercial insurance plans,^10^ we decided a priori to perform models stratified by insurance type (subgroup 1: private only and both private and public; subgroup 2: public only, uninsured, and unknown). After finding no substantial differences in stratified models, we analyzed the full sample in final models. There was a missing value for at least 1 variable used in the multivariable models for 1636 of 29 876 caregivers (5.5%) in the sample. Final models used the 95% of the sample with complete data.

An additional sensitivity analysis that removed respondents who reported that their child or adolescent did not need MBH care was conducted. The association between SMHILD and perceived adequacy of insurance remained the same (eTable 3 in Supplement 1).

All effect estimates were reported as adjusted odds ratios (aORs) with 95% CIs. Statistical tests were 2-tailed, and a *P* value was set at <.05. Data analyses were performed using Stata statistical software version 16.0 (StataCorp).

## Results

### Characteristics of Study Participants

During the 4-year period, we identified 29 876 caregiver respondents with children or adolescents who had MBH conditions, which represented 14 292 300 children and adolescents nationally (6 475 573 aged 6-11 years [45.3%] and 7 816 727 aged 12-17 years [54.7%]; 8 455 171 male [59.2%]; 292 543 Asian [2.0%], 2 076 442 Black [14.5%], and 9 942 088 White [69.6%]; 3 202 525 Hispanic [22.4%]) (Table 1). An estimated 5 195 426 children and adolescents lived in a state with a SMHILD score of 4, representing the most common score among participants (eTable 4 in Supplement 1). A maximally comprehensive parity score of 7 was achieved by 1 state.

**Table 1.** Characteristics of Study Population

Characteristic	Survey respondents, No. (N = 29 876)	Population estimate of children and adolescents with MBH condition, No. (%) (N = 14 292 300)
Age, y		
6-11	11 406	6 475 573 (45.3)
12-17	18 470	7 816 727 (54.7)
Sex		
Male	17 351	8 455 171 (59.2)
Female	12 525	5 837 129 (40.8)
Race		
American Indian or Alaska Native	286	159 919 (1.1)
Asian	732	292 543 (2.0)
Black or African American	1996	2 076 442 (14.5)
Native Hawaiian and Other Pacific Islander	89	102 512 (0.7)
White	24 098	9 942 088 (69.6)
Other^a^	479	525 243 (3.7)
≥2 Races	2196	1 193 554 (8.4)
Ethnicity		
Hispanic or Latino origin	3181	3 202 525 (22.4)
Not Hispanic or Latino origin	26 695	11 089 776 (77.6)
Primary household language		
No. with data	29 714	NA
English	28 820	13 016 188 (91.8)
Spanish	568	917 347 (6.5)
Other	326	242 529 (1.7)
Insurance coverage		
No. with data	29,454	NA
Public only	7364	4 966 501 (35.5)
Private only or private and public	21 032	8 296 132 (59.3)
Not insured	1058	726 850 (5.2)
Child or adolescent place of birth		
No. with data	29 732	NA
US	28 822	13 712 256 (96.4)
Outside the US	910	505 578 (3.6)
Family structure		
No. with data	29 371	NA
2 Caregivers, married	19 088	8 100 115 (58.3)
2 Caregivers, not married	1936	1 219 462 (8.8)
1 Caregiver	6119	3 362 260 (24.2)
Other	2228	1 218 627 (8.8)
Primary caretaker place of birth		
No. with data	29,337	NA
US	27 179	12 075 618 (86.9)
Outside the US	2158	1 822 882 (13.1)
Primary caretaker educational attainment		
No. with data	29 532	NA
<High school	1076	1 876 119 (13.3)
High school, GED, vocational, or trade	5309	3 341 106 (23.6)
Some college	4815	2 029 838 (14.4)
Associate degree	3345	1 286 536 (9.1)
Bachelor’s degree	8287	3 149 283 (22.3)
Advanced degree (master’s or doctorate)	6700	2 453 370 (17.4)
Primary caretaker mental and emotional health		
No. with data	29 240	NA
Excellent	7970	3 811 168 (27.5)
Very good	11 838	5 216 008 (37.7)
Good	7076	3 490 376 (25.2)
Fair or poor	2356	1 328 875 (9.6)
ACE score sum		
No. with data	29 512	NA
0	12 454	5 339 429 (38.1)
1	7114	3 483 236 (24.9)
2	3952	1 944 289 (13.9)
3	2363	1 263 307 (9.0)
≥4	3629	1 966 118 (14.0)
Child or adolescent seen by medical clinician (past 12 mo)		
No. with data	29 848	NA
Yes	26 804	12 283 242 (86.1)
No	3 044	1 977 144 (13.9)
General description of child or adolescent health		
No. with data	29 785	NA
Excellent	14 441	6 482 948 (45.5)
Very good	9996	4 698 324 (33.0)
Good	4310	2 462 759 (17.3)
Fair or poor	1038	613 753 (4.3)
Needed MBH services not received		
No. with data	1780	NA
Yes	914	541 411 (49.4)
No	866	554 494 (50.6)
MBH treatment or counseling received		
No. with data	29 743	NA
Yes	11 228	4 898 603 (34.5)
No, but this child needed to see a MH professional	1587	951 987 (6.7)
No, this child did not need to see a MH professional	16 928	8 354 474 (58.8)
Problem or difficulty getting needed MH treatment or counseling		
No. with data	29 586	NA
Not a problem or not difficult	7145	3 054 757 (21.6)
Small problem or somewhat difficult	3608	1 737 088 (12.3)
Very difficult or It was not possible to obtain care	1905	979 994 (6.9)
This child did not need MH care	16 928	8 354 474 (59.1)
Perceived poor access to MBH care^b^		
Yes	3193	1 770 492 (12.4)
No	26 683	12 521 809 (87.6)
Perceived adequacy of mental and behavioral health insurance coverage^c^		
No. with data	28 238	NA
Always	7657	3 434 233 (26.1)
Usually	4350	1 906 090 (14.5)
Sometimes	2481	1 079 410 (8.2)
Never	1036	563 850 (4.3)
Child does not use mental or behavioral health services	12 714	6 191 712 (47.0)

Abbreviations: ACE, adverse childhood experience; GED, General Educational Development; MBH, mental and behavioral health; MH, mental health; NA, not applicable.

^a^ American Indian or Alaska Native alone, Native Hawaiian or Other Pacific Islander alone, or other race alone.

^b^ This is a composite outcome as described in the methods.

^c^ Observations with “not insured” for insurance coverage status were excluded.

### Access to Mental and Behavioral Health Care

A total of 3193 caregivers representing 1 770 492 children and adolescents (12.4% nationally) perceived that their child or adolescent had poor access to MBH care. Perceived poor access to MBH care occurred most frequently among caregivers of children and adolescents living in states with SMHLID scores of 0 to 2 (13.5%, representing 406 034 of 3 008 939 children and adolescents nationally) and 4 (12.9%, representing 671 520 of 5 195 426 children and adolescents nationally) (Table 2; Figure). In the multivariable model, caregivers of children and adolescents living in the 12 states with the most comprehensive MBH insurance legislation (scores, 5-7) had decreased odds of perceiving poor access to MBH care (aOR, 0.79; 95% CI, 0.63-0.99) compared with caregivers of children and adolescents living in the 10 states with the least comprehensive insurance legislation (scores, 0-2). Adjusted proportions of perceived poor access to MBH care were 10.5% (95% CI, 9.1%-11.9%) and 12.8% (95% CI, 11.1%-14.5%) in the most and least comprehensive MBH insurance legislation groups, respectively.

**Table 2.** Perceived MBH Access and Insurance Adequacy by Comprehensiveness of State Laws

Caregiver perception	Children and adolescents living in state, No. (%)^a^			
	Score: 0-2	Score: 3	Score: 4	Score: 5-7
Perceived poor access to mental and behavioral health care				
Total population-level estimate, No.	3 008 939^b^	3 534 276^b^	5 195 426^b^	2 553 658^b^
No	2 602 905 (86.5)	3 114 535 (88.1)	4 523 906 (87.1)	2 280 463 (89.3)
Yes	406 034 (13.5)	419 741 (11.9)	671 520 (12.9)	273 195 (10.7)
Perceived adequacy of mental and behavioral health insurance coverage				
Total population-level estimate, No.	2 777 526^c^	3 261 686^c^	4 768 393^c^	2 367 689^c^
Always	794 902 (28.6)	894 559 (27.4)	1 109 814 (23.3)	634 958 (26.8)
Usually	389 100 (14.0)	471 611 (14.5)	703 470 (14.8)	341 909 (14.4)
Sometimes	246 203 (8.9)	229 669 (7.0)	434 093 (9.1)	169 445 (7.2)
Never	92 519 (3.3)	125 626 (3.9)	270 221 (5.7)	75 483 (3.2)
Child does not use MBH services	1 254 802 (45.2)	1 540 221 (47.2)	2 250 795 (47.2)	1 145 894 (48.4)

Abbreviation: MBH, mental and behavioral health.

^a^ Comprehensiveness of state insurance laws was measured by the State Mental Health Insurance Laws Dataset score.

^b^ Population-level estimates over study period, entire sample.

^c^ Population-level estimates over study period; observations with “not insured” for insurance coverage status were excluded.

**Figure.**
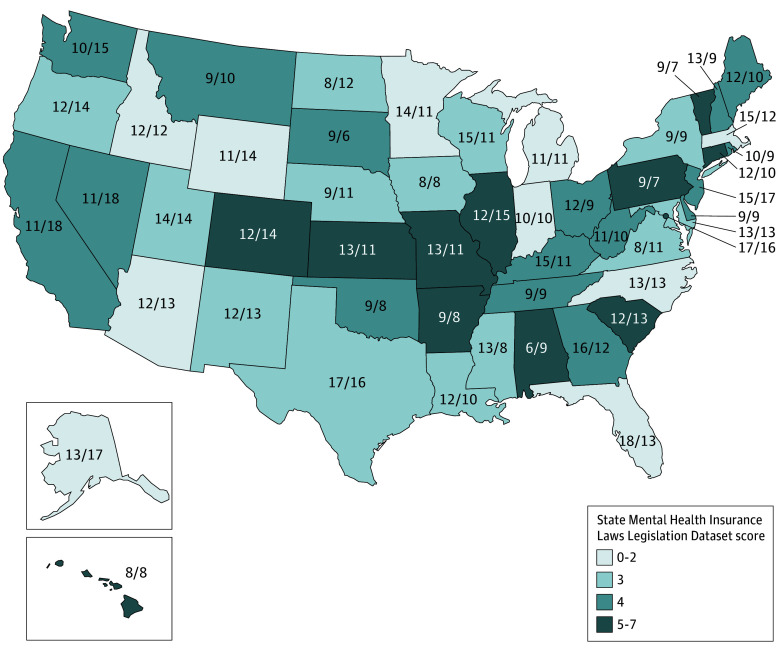
State Mental Health Insurance Laws and Perceived Access and Coverage The percentage of caregivers who perceived poor access to mental and behavioral health care and inadequate mental and behavioral health insurance coverage are reflected by numbers within each state. The color of the state represents the State Mental Health Insurance Laws Dataset score (0-7).

Compared with caregivers of White children and adolescents, there were higher adjusted odds of perceived poor access to MBH care among caregivers of Black or African American (aOR, 1.35; 95% CI, 1.04-1.75) and Asian (aOR, 1.69; 95% CI, 1.01-2.84) children and adolescents (Table 3). Compared with children and adolescents with no ACE exposures, the adjusted odds of perceived poor access to MBH care increased as the number of ACE exposures among children and adolescents increased (1 ACE: aOR, 1.68; 95%, CI 1.32-2.13; 2 ACEs: aOR, 2.19; 95% CI, 1.66-2.88; 3 ACEs: aOR, 3.02; 95% CI, 2.19-4.16; ≥4 ACEs: aOR, 4.28; 95% CI, 3.17-5.77).

**Table 3.** Association of Comprehensiveness of State Laws With Perceived MBH Access and Insurance Coverage

Factor	aOR (95% CI)	
	Perceived poor access to MBH care (n = 29 876)	Perceived inadequate MBH insurance coverage (n = 28 396)^a^
Parity score^b^		
Lowest (0-2)	1 [Reference]	1 [Reference]
Second (3)	0.87 (0.69-1.09)	0.90 (0.74-1.10)
Third (4)	1.02 (0.81-1.28)	1.23 (1.01-1.49)
Highest (5-7)	0.79 (0.63-0.99)	0.85 (0.70-1.04)
Age, y		
6-11	1 [Reference]	1 [Reference]
12-17	1.04 (0.87-1.25)	0.94 (0.80-1.10)
Sex		
Male	1 [Reference]	1 [Reference]
Female	1.05 (0.89-1.24)	1.03 (0.88-1.21)
Race^c^		
Asian	1.69 (1.01-2.84)	1.15 (0.72-1.84)
Black or African American	1.35 (1.04-1.75)	0.86 (0.64-1.15)
White	1 [Reference]	1 [Reference]
≥2 Races	0.70 (0.52-0.94)	0.82 (0.61-1.10)
Other^d^	1.01 (0.63-1.64)	1.06 (0.61-1.83)
Ethnicity		
Hispanic or Latino origin	1 [Reference]	1 [Reference]
Not Hispanic or Latino origin	1.03 (0.77-1.38)	0.98 (0.72-1.33)
Primary household language		
English	1 [Reference]	1 [Reference]
Spanish	2.07 (1.02-4.17)	1.66 (0.90-3.05)
Other	1.41 (0.74-2.66)	1.37 (0.75-2.49)
Insurance coverage^a^		
Public only	1 [Reference]	1 [Reference]
Private only or private and public	1.06 (0.87-1.30)	1.62 (1.28-2.04)
Not insured	1.65 (1.11-2.44)	NA
Child or adolescent place of birth		
US	1 [Reference]	1 [Reference]
Outside the US	1.33 (0.83-2.13)	0.97 (0.64-1.48)
Family structure		
2 Caregivers, married	1 [Reference]	1 [Reference]
2 Caregivers, not married	1.10 (0.79-1.52)	1.11 (0.80-1.54)
1 Caregiver	0.98 (0.80-1.21)	0.97 (0.78-1.21)
Other	0.93 (0.67-1.30)	0.75 (0.51-1.10)
Primary caretaker place of birth		
US	1 [Reference]	1 [Reference]
Outside the US	0.73 (0.46-1.15)	1.01 (0.72-1.43)
Primary caretaker educational attainment		
<High school	0.71 (0.48-1.05)	0.85 (0.51-1.41)
High school, GED, vocational, or trade	1 [Reference]	1 [Reference]
Some college	1.15 (0.89-1.49)	1.27 (0.98-1.66)
Associate degree	1.02 (0.72-1.43)	1.28 (0.93-1.75)
Bachelor’s degree	1.16 (0.90-1.50)	1.82 (1.42-2.33)
Advanced degree (master’s or doctorate)	1.49 (1.15-1.92)	2.38 (1.84-3.06)
Primary caretaker mental and emotional health		
Excellent	1 [Reference]	1 [Reference]
Very good	1.30 (0.99-1.71)	1.15 (0.95-1.40)
Good	1.56 (1.17-2.08)	1.32 (1.04-1.66)
Fair or poor	2.41 (1.70-3.41)	1.83 (1.32-2.54)
ACE score sum		
0	1 [Reference]	1 [Reference]
1	1.68 (1.32-2.13)	1.05 (0.86-1.28)
2	2.19 (1.66-2.88)	1.20 (0.91-1.57)
3	3.02 (2.19-4.16)	1.24 (0.91-1.70)
≥4	4.28 (3.17-5.77)	1.53 (1.13-2.08)
Child or adolescent seen by medical clinician (past 12 mo)		
No	1 [Reference]	1 [Reference]
Yes	0.88 (0.66-1.16)	0.84 (0.63-1.12)
General description of child or adolescent health		
Excellent	1 [Reference]	1 [Reference]
Very good	1.56 (1.26-1.95)	1.32 (1.09-1.58)
Good	1.91 (1.48-2.47)	1.48 (1.14-1.93)
Fair or poor	2.69 (1.88-3.84)	2.01 (1.41-2.88)

Abbreviations: ACE, adverse childhood experience; aOR, adjusted odds ratio; GED, General Educational Development; MBH, mental and behavioral health; NA, not applicable.

^a^ Observations with “not insured” for insurance coverage status were excluded.

^b^ Comprehensiveness of state insurance laws was measured by the State Mental Health Insurance Laws Dataset score.

^c^ White is used as the reference group because it was the largest group in the study population.

^d^ Other race includes American Indian or Alaska Native alone, Native Hawaiian or Other Pacific Islander alone, or other race alone.

### Perceived Adequacy of MBH Insurance Coverage

A total of 3517 caregivers representing 1 643 260 of 13 175 295 children and adolescents nationally (12.5%) perceived that their child or adolescent had inadequate MBH insurance coverage. Perceived inadequacy of MBH insurance coverage occurred most frequently among caregivers of children and adolescents living in states with SMHILD scores of 4 (14.8%, representing 704 314 of 4 768 393 children and adolescents nationally) (Table 2). In the multivariable model, caregivers of children and adolescents living in the 16 states with moderately comprehensive MBH insurance legislation (score, 4) had increased odds of perceived inadequate MBH insurance coverage (aOR, 1.23; 95% CI, 1.01-1.49) compared with caregivers of children and adolescents living in the 10 states with the least comprehensive legislation (score, 0-2). Adjusted proportions of perceived inadequate MBH insurance coverage were 14.3% (95% CI, 12.7%-16.0%) and 12.1% (95% CI, 10.6%-13.5%) in moderate (score, 4) and least (score, 0-2) comprehensive MBH insurance legislation groups, respectively. Caregivers of children and adolescents with 4 or more ACEs had higher adjusted odds of perceived inadequate MBH insurance coverage compared with caregivers of children and adolescents with no ACEs (aOR, 1.53; 95% CI, 1.13-2.08).

## Discussion

In this nationally representative cross-sectional study, we found that caregivers representing approximately 1 in 8 children and adolescents with MBH conditions perceived that they had poor access to MBH care (12.4%) and inadequate MBH insurance coverage (12.5%). Caregivers of children and adolescents living in states with highly comprehensive laws had approximately 20% lower odds of perceived poor access to MBH care compared with caregivers of children and adolescents living in states with the least comprehensive state mental health insurance laws. This association was consistent regardless of insurance type. Unexpectedly, caregivers of children and adolescents in states with a moderate SMHILD score (4) had nearly 25% higher likelihood of perceived inadequate MBH insurance coverage than caregivers of children and adolescents living in states with the least comprehensive state mental health insurance laws.

These findings introduce the idea that state parity legislation may be associated with access to child and adolescent MBH services in the US.^32^ However, the adoption, implementation, and enforcement of mental health parity laws varies widely by state.^33,34^ Prior studies have found that families of children and adolescents with MBH conditions who live in states with parity laws experience significantly reduced annual out-of-pocket costs for MBH care compared with families living in states without such laws.^35,36^ Thus, parity laws may be associated with the immediate affordability of care. Additionally, increased access to care conferred by parity laws may have long-term health benefits. In a longitudinal study,^15^ exposure to more comprehensive state mental health parity legislation during adolescence was associated with lower rates of MBH care use in adulthood.

We were surprised to find increased odds of perceived inadequate health insurance among caregivers of children and adolescents living in states with moderately comprehensive insurance laws. The reasons for this finding are likely multifactorial. Certain elements of the score may have greater influence on perceived adequacy of health insurance, which are shared by states with moderately comprehensive scores; further study is needed to assess score components. Additionally, unmeasured child-level and state-level confounders may be associated with perceptions of health insurance adequacy. As a possible child or adolescent–level factor, even when families have insurance, MBH clinicians may refuse to accept certain insurance types or insurance altogether, contributing to perceived inadequate coverage.^37^ Additionally, even when children and adolescents are insured, caregivers may be adversely impacted by cost-sharing for child or adolescent MBH care.^38^ For states with legislation mandating parity, the quality of parity enforcement likely varies by staffing capacity and resource availability to conduct in-depth assessments and evaluations.^39,40^ Furthermore, relationships between state insurance offices and other parties (eg, legislators and advocacy groups) may influence prioritization of state-level parity enforcement.^41^ Perceptions of MBH insurance adequacy may also be influenced by the extent to which insurers and states educate the public about parity laws and violations. Caregivers who lack awareness of mandated benefits may be less likely to recognize violations of parity mandates. Delivering education about insurance coverage and state-specific parity laws in multiple languages at appropriate reading levels may reduce barriers to accessing MBH care and reduce out-of-pocket caregiver spending.^42^

We found that perceived access to MBH care varied substantially by individual-level factors. Specifically, perceptions of access to MBH care differed by race, ethnicity, household language, caregiver education, perceived health of the child or adolescent or the caregiver, and child or adolescent ACE exposure, reflecting inequities in access to MBH care across population groups.^43,44^ Although some MBH conditions are more prevalent among minoritized racial and ethnic groups,^45^ these children and adolescents are less likely to use MBH services, such as MBH specialty visits and psychotropic medication prescriptions, resulting in lower MBH expenditures.^27,46,47,48,49^ These inequities may contribute to adverse health outcomes in these populations, such as increased rates of physical and pharmacologic restraint for acute agitation espisodes,^50,51^ and increasing suicide rates.^52,53^ However, state-level parity legislation may be associated with individual-level disparities, with stronger laws associated with narrowed differences across demographic or socioeconomic subgroups.^15^ Additional policy-level solutions to improve access to MBH services across racial and ethnic groups include investing in a language- and culture-concordant MBH professional workforce, expanding community-based MBH services, and supporting payment structures that enable access to tele–mental health care.^27^

We found that caregivers of children and adolescents who experienced more ACEs were more likely to perceive that they had poor access to MBH care, in a dose-dependent fashion. ACEs are traumatic events, such as maltreatment or abuse, that can have lasting effects on child health and well-being.^4^ Cumulative effects of ACEs over time include chronic mental and physical health conditions, increased suicide risk, and substance use disorders.^54,55,56,57^ Consistent with our findings on access to MBH care, prior studies found that increased ACE exposure was associated with lower health care use.^54,55,56,57,58^ Although screening for ACEs is recommended by the American Academy of Pediatrics,^58^ screening has not been associated with improved MBH outcomes for children.^59^ However, screening may prompt referrals to early intervention programs, such as Head Start and Nurse-Family Partnership, which support psychological resilience, promote positive childhood experiences, and improve family functioning for children who have experienced ACEs.^60,61^

Our findings have implications for policymakers interested in legislative strategies to improve child and adolescent access to MBH care. It is striking that during the study period, only 1 state achieved a maximally comprehensive parity score of 7, encompassing themes of parity, mandated coverage, mental health condition definition, and enforcement-compliance. This highlights opportunities for nearly all states to enhance comprehensiveness of MBH parity legislation. Prior work has found that specific elements of parity legislation, such as laws regarding enforcement and compliance, are uncommonly included in state legislature.^10^ Additionally, some states miss opportunities to define mental health conditions in parity laws in ways that are relevant for children and adolescents. For instance, definitions may not include child and adolescent MBH disorders listed in the *Diagnostic and Statistical Manual of Mental Disorders*, such as autism spectrum disorder and attention-deficit/hyperactivity disorder.^62,63^ State legislators should ensure that definitions of mental health conditions in parity laws include childhood-relevant disorders, so that children and adolescents with MBH conditions can access needed care. Legislation related to mental health parity is one of many policy approaches that can be considered to improve access to mental health services for children and adolescents; policies are also needed to promote MBH integration in primary care, access to telehealth, and expansion of the MBH professional workforce.^64,65,66,67^

### Limitations

This study has several limitations. The NSCH relies on caregiver self-report, which may be subject to recall bias and social desirability limitations, particularly in reporting MBH conditions. Additionally, the NSCH uses address-based sampling; therefore, findings may not be generalizable to children and adolescents and caregivers who do not have legal immigration status, who have housing instability, or who reside in foster care or congregate care settings. The datasets used do not characterize state-level differences in MBH clinician supply, which may also influence perception of access to MBH care and is an important area for future work. Additionally, the SMHILD includes only legislation passed by state legislatures, although some jurisdictions have addressed mental health insurance parity through administrative regulations.

## Conclusions

In this cross-sectional study, comprehensiveness of state mental health insurance legislation was associated with perceived access to MBH care. Specifically, we found that caregivers of children and adolescents living in states with the most comprehensive state mental health parity legislation had a lower likelihood of perceived access barriers. However, caregivers of children and adolescents living in states with a moderate comprehensiveness score had increased odds of perceived inadequacy of MBH insurance coverage. Further study is needed to understand what elements of comprehensive mental health parity legislation are associated with patient-level outcomes for US children and adolescents with MBH conditions. Nevertheless, state mental health parity laws may be one lever policymakers can use to improve access to care for children and adolescents with MBH conditions.

## Notes

Supplement 1. **eTable 1.** The State Mental Health Insurance Laws Dataset Legal Coding Instrument **eTable 2.** Definition of Mental and Behavioral Health Condition **eTable 3.** Sensitivity Analysis With Removal of Respondents Who Reported Their Child or Adolescent Did Not Need Mental and Behavioral Health Care **eTable 4.** Population Characteristics of Children and Adolescents With Mental and Behavioral Health Conditions by State Mental Health Insurance Laws Dataset, 2016-2019

Supplement 2.
Data Sharing Statement
