# Supplementary material for: Comprehensiveness of State Insurance Laws and Perceived Access to Pediatric Mental Health Care
Source: JAMA Netw Open. 2024 Aug 12;7(8):e2426402. doi: 10.1001/jamanetworkopen.2024.26402 (PMC11320173; doi:10.1001/jamanetworkopen.2024.26402)
Supplement: Supplement 1. — eTable 1. The State Mental Health Insurance Laws Dataset Legal Coding Instrument eTable 2. Definition of Mental and Behavioral Health Condition eTable 3. Sensitivity Analysis With Removal of Respondents Who Reported Their Child or Adolescent Did Not Need Mental and Behavioral Health Care eTable 4. Population Characteristics of Children and Adolescents With Mental and Behavioral Health Conditions by State Mental Health Insurance Laws Dataset, 2016-2019 [file jamanetwopen-e2426402-s001.pdf]

Supplemental Online Content

Foster AA, Hoffmann JA, Douglas MD, et al. Comprehensiveness of state insurance laws and perceived access to pediatric mental health care. *JAMA Netw Open*. 2024;7(8):e2426402. doi:10.1001/jamanetworkopen.2024.26402

eTable 1. The State Mental Health Insurance Laws Dataset Legal Coding Instrument
eTable 2. Definition of Mental and Behavioral Health Condition
eTable 3. Sensitivity Analysis With Removal of Respondents Who Reported Their Child or Adolescent Did Not Need Mental and Behavioral Health Care
eTable 4. Population Characteristics of Children and Adolescents With Mental and Behavioral Health Conditions by State Mental Health Insurance Laws Dataset, 2016-2019

This supplemental material has been provided by the authors to give readers additional information about their work.

© 2024 Foster AA et al. *JAMA Netw Open*.

eTable 1. The State Mental Health Insurance Laws Dataset Legal Coding Instrument

**Level of Parity**	**Range of points assigned**
Does a state statute require that coverage provided for treatment of mental health conditions must be on the same terms and conditions as it is for other medical coverage?	0,1,2
**Mandate in place**	
Does a state statute mandate health insurance / benefit plans to provide coverage for treatment of mental health conditions?	0,1
**Definition of mental health condition**	
Does a relevant state statute define mental illness or mental health conditions?	0,1
Does a state statute define mental illness or mental health conditions as including all of the disorders listed in the Diagnostic and Statistical Manual of Mental Disorders (DSM) or International Classification of Diseases (ICD)?	0,1
**Enforcement and Compliance**	
Does a state statute require the state insurance department or other relevant state agency to enforce or implement the Federal Parity Law or state parity law?	0,1
Does a state statute require health insurance/benefit plans to submit reports to the state insurance department or other relevant state agency demonstrating how they comply with the Federal Parity Law and/or any state parity statutes or regulations?	0,1

© 2024 Foster AA et al. *JAMA Netw Open.*

eTable 2. Definition of Mental and Behavioral Health Condition

**National Survey of Children's Health Questions and Response Options**[1]
Has a doctor or other healthcare provider ever told you that this child has…Anxiety Problems?
Has a doctor or other healthcare provider ever told you that this child has…Depression?
Has a doctor or other healthcare provider, or educator ever told you that this child has…Behavioral or Conduct problems?
Has a doctor or other health care provider ever told you that this child has…Substance Use Disorder?
Has a doctor or other health care provider ever told you that this child has Autism or Autism spectrum disorder (ASD)?
Has a doctor or other health care provider ever told you that this child has…Attention Deficit Disorder or Attention Deficit/Hyperactivity Disorder, that is, ADD or ADHD?
Has a doctor or other health care provider ever told you that this child has…Tourette Syndrome?
Has a doctor or other health care provider ever told you that this child has…Developmental Delay?
Has a doctor or other health care provider ever told you that this child has…Intellectual Disability?
Has a doctor or other health care provider ever told you that this child has…Speech or other language disorder?
Has a doctor or other health care provider ever told you that this child has… Learning Disability?
Has a doctor or other health care provider ever told you that this child has… Any other mental health condition?

[1]We considered a child to have a mental or behavioral health condition if the caregiver answered yes to any of the questions listed.

© 2024 Foster AA et al. *JAMA Netw Open.*

eTable 3. Sensitivity Analysis with Removal of Respondents who Reported Their Child Did Not Need Mental and Behavioral Health Care

Predictor	Perceived inadequate mental and behavioral health insurance coverage, aOR (95% CI) (n=15,682) [1]
**Parity Score**	
Lowest (0-2)	referent
Second (3)	0.92 (0.75, 1.14)
Third (4)	**1.30 (1.06, 1.60)**
Highest (5-7)	0.89 (0.72, 1.10)
**Age (years)**	
6-11	referent
12-17	**0.84 (0.70, 0.99)**
**Sex**	
Male	referent
Female	1.04 (0.88, 1.23)
**Race**	
White	referent
Black or African American	0.91 (0.67, 1.23)
Asian	1.15 (0.68, 1.95)
Two or more races	0.84 (0.62, 1.14)
Other[1]	1.31 (0.74, 2.33)
**Ethnicity**	
Hispanic or Latino origin	referent
Not Hispanic or Latino origin	0.95 (0.69, 1.30)
**Primary Household Language**	
English	referent
Spanish	1.59 (0.82, 3.09)
Other	1.50 (0.76, 2.98)
**Insurance Coverage**	
Public only	referent
Private only/Private and public	**1.69 (1.32, 2.16)**
**Child place of birth**	
In the United States	referent
Outside the United States	0.79 (0.47, 1.33)
**Family Structure**	
Two caregivers, married	referent

© 2024 Foster AA et al. *JAMA Netw Open.*

Two caregivers not married	1.11 (0.79, 1.56)
One caregiver	0.93 (0.74, 1.17)
Other	**0.65 (0.44, 0.95)**
**Primary caretaker place of birth**	
In the United States	referent
Outside the United States	1.04 (0.72, 1.51)
**Primary caretaker educational attainment**	
Less than high school	0.75 (0.44, 1.28)
High school/GED/Vocational/Trade	referent
Some college	1.23 (0.93, 1.64)
Associate degree	1.23 (0.88, 1.72)
Bachelor's degree	**1.56 (1.20, 2.04)**
Advanced degree (masters or doctorate)	**1.95 (1.49, 2.57)**
**Primary caretaker mental/emotional health**	
Excellent	referent
Very good	1.01 (0.82, 1.25)
Good	1.08 (0.85, 1.39)
Fair/poor	**1.47 (1.04, 2.08)**
**Adverse Childhood Experience Score Sum**	
0	referent
1	0.93 (0.75, 1.15)
2	0.94 (0.71, 1.25)
3	0.98 (0.71, 1.37)
≥4	1.08 (0.78, 1.48)
**Child seen by medical clinician (past 12 months)**	
No	referent
Yes	0.77 (0.56, 1.05)
**General description of child's health**	
Excellent	referent
Very good	1.07 (0.88, 1.29)
Good	1.07 (0.81, 1.41)
Fair/poor	1.40 (0.96, 2.06)

Abbreviations: GED, General Educational Development

[1] Observations with "not insured" for Insurance Coverage status excluded; observations reporting that the child did not need MH care excluded

[2] American Indian or Alaska Native alone, Native Hawaiian or Other Pacific Islander alone, other race alone

© 2024 Foster AA et al. *JAMA Netw Open*.

eTable 4. Population Characteristics of Children with Mental and Behavioral Health Conditions by State Mental Health Insurance Laws Dataset, 2016-2019

Patient Characteristics	State-Level Parity Score			
	**0-2**	**3**	**4**	**5-7**
**Overall**	n=3,008,939 **(%)**	n=3,534,276 **(%)**	n=5,195,426 **(%)**	n=2,553,659 **(%)**
**Age (years)**				
6-11	1,324,712 (44.0)	1,579,289 (44.7)	2,357,859 (45.4)	1,213,713 (47.5)
12-17	1,684,227 (56.0)	1,954,987 (55.3)	2,837,567 (54.6)	1,339,946 (52.5)
**Sex**				
Male	1,765,953 (58.7)	2,117,999 (60.0)	3,055,845 (58.8)	1,515,375 (59.3)
Female	1,242,987 (41.3)	1,416,277 (40.1)	2,139,582 (41.2)	1,038,284 (40.7)
**Race**				
White	2,250,984 (74.8)	2,373,155 (67.1)	3,500,440 (67.4)	1,817,508 (71.2)
Black or African American	401,674 (13.3)	638,727 (18.1)	605,863 (11.7)	430,178 (16.8)
American Indian or Alaska Native	29,058 (1.0)	28,708 (0.8)	80,887 (1.6)	21,266 (0.8)
Asian	43,017 (1.4)	56,569 (1.6)	146,179 (2.8)	46,778 (1.8)
Native Hawaiian and other Pacific Islander	12,053 (0.4)	15,055 (0.4)	47,659 (0.9)	27,745 (1.1)
Other[1]	65,881 (2.2)	163,508 (4.6)	236,457 (4.6)	59,396 (2.3)
Two or more races	206,272 (6.9)	258,553 (7.3)	577,941 (11.1)	150,788 (5.9)
**Ethnicity**				
Hispanic or Latino origin	517,750 (17.2)	769,986 (21.8)	1,572,253 (30.3)	342,535 (13.4)
Not Hispanic or Latino origin	2,491,189 (82.8)	2,764,290 (78.2)	3,623,173 (69.7)	2,211,124 (86.6)
**Primary Household Language**				
English	2,748,247 (91.3)	3,232,204 (91.5)	4,593,131 (88.4)	2,442,606 (95.7)
Spanish	162,695 (5.4)	210,226 (5.9)	482,614 (9.3)	61,812 (2.4)
Other	65,443 (2.2)	56,319 (1.6)	82,730 (1.6)	38,037 (1.5)
**Insurance Coverage**				
Public only	1,109,250 (36.9)	1,158,023 (32.8)	1,786,963 (34.4)	912,266 (35.7)
Private only/Private and public	1,692,292 (56.2)	2,129,960 (60.3)	3,007,210 (57.9)	1,466,669 (57.4)
Not insured	141,853 (4.7)	187,488 (5.3)	277,873 (5.3)	119,636 (4.7)
**Child place of birth**				
In the United States	2,868,316 (95.3)	3,421,791 (96.8)	4,977,293 (95.8)	2,444,857 (95.7)
Outside the United States	129,977 (4.3)	94,264 (2.7)	193,449 (3.7)	87,887 (3.4)

© 2024 Foster AA et al. *JAMA Netw Open.*

**Family Structure**				
Two caregivers, married	1,641,838 (54.6)	2,027,981 (57.4)	3,026,848 (58.3)	1,403,447 (55.0)
Two caregivers not married	281,797 (9.4)	269,016 (7.6)	449,071 (8.6)	219,579 (8.6)
One caregiver	714,029 (23.7)	828,558 (23.4)	1,181,015 (22.7)	638,659 (25.0)
Other	276,734 (9.2)	317,626 (9.0)	389,709 (7.5)	234,558 (9.2)
**Primary caretaker place of birth**				
In the United States	2,613,983 (86.9)	3,002,618 (85.0)	4,143,698 (79.8)	2,315,319 (90.7)
Outside the United States	307,684 (10.2)	435,170 (12.3)	907,888 (17.5)	172,139 (6.7)
**Primary caretaker educational attainment**				
Less than high school	367,868 (12.2)	481,878 (13.6)	766,146 (14.7)	260,227 (10.2)
High school/GED/Vocational/Trade	683,066 (22.7)	848,796 (24.0)	1,149,016 (22.1)	660,228 (25.9)
Some college	471,415 (15.7)	481,237 (13.6)	725,893 (14.0)	351,292 (13.8)
Associate degree	277,985 (9.2)	308,120 (8.7)	452,024 (8.7)	248,407 (9.7)
Bachelor's degree	650,942 (21.6)	758,791 (21.5)	1,175,293 (22.6)	564,257 (22.1)
Advanced degree (masters or doctorate)	508,994 (16.9)	620,625 (17.6)	874,370 (16.8)	449,382 (17.6)
**Primary caretaker mental/emotional health**				
Excellent	725,925 (24.1)	1,050,935 (29.7)	1,405,410 (27.1)	628,898 (24.6)
Very good	1,183,168 (39.3)	1,242,518 (35.2)	1,833,384 (35.3)	956,939 (37.5)
Good	701,408 (23.3)	792,297 (22.4)	1,338,359 (25.8)	658,313 (25.8)
Fair/poor	293,642 (9.8)	337,108 (9.5)	460,393 (8.9)	237,732 (9.3)
**Adverse Childhood Experience Score Sum**				
0	1,070,647 (35.6)	1,298,296 (36.7)	2,020,343 (38.9)	950,143 (37.2)
1	676,917 (22.5)	957,549 (27.1)	1,212,115 (23.3)	636,656 (24.9)
2	408,644 (13.6)	480,754 (13.6)	703,077 (13.5)	351,814 (13.8)
3	278,636 (9.3)	280,342 (7.9)	470,064 (9.0)	234,264 (9.2)
≥4	504,835 (17.0)	418,926 (11.9)	697,380 (13.4)	344,976 (13.5)
**Child seen by medical clinician (past 12 months)**				
Yes	2,609,969 (86.7)	3,005,642 (85.0)	4,406,090 (84.8)	2,261,541 (88.6)
No	390,356 (13.0)	524,559 (14.8)	771,942 (14.9)	290,286 (11.4)
**General description of child's health**				
Excellent	1,354,921 (45.0)	1,627,952 (46.1)	2,340,779 (45.1)	1,159,296 (45.4)
Very good	994,768 (33.1)	1,097,092 (31.0)	1,737,347 (33.4)	869,117 (34.0)
Good	499,862 (16.6)	614,634 (17.4)	931,409 (17.9)	416,855 (16.3)
Fair/poor	152,924 (5.1)	186,301 (5.3)	176,512 (3.4)	98,017 (3.8)
**How much of a problem was it to get the MH treatment or counseling that this child needed?**				

© 2024 Foster AA et al. *JAMA Netw Open*.

Not a problem/Not difficult	683,202 (22.7)	715,606 (20.2)	1,089,759 (21.0)	566,191 (22.2)
Small problem/somewhat difficult	345,372 (11.5)	445,980 (12.6)	660,473 (12.7)	285,262 (11.2)
Very difficult + It was not possible to obtain care	254,425 (8.5)	218,043 (6.2)	345,343 (6.6)	162,184 (6.4)
This child did not need MH care	1,699,468 (56.5)	2,112,730 (59.8)	3,035,018 (58.4)	1,507,258 (59.0)
**Perceived poor access to MBH care**[2]				
No	2,602,905 (86.5)	3,114,535 (88.1)	4,523,906 (87.1)	2,280,463 (89.3)
Yes	406,034 (13.5)	419,741 (11.9)	671,520 (12.9)	273,195 (10.7)
**Perceived adequacy of MH insurance coverage**[3]				
Always	814,901 (27.1)	905,632 (25.6)	1,134,493 (21.8)	649,160 (25.4)
Usually	393,418 (13.1)	479,510 (13.6)	716,902 (13.8)	346,878 (13.6)
Sometimes	248,431 (8.3)	232,407 (6.6)	445,648 (8.6)	170,708 (6.7)
Never	94,931 (3.2)	141,164 (4.0)	284,390 (5.5)	79,089 (3.1)
Child does not use MH services	1,281,444 (42.6)	1,560,630 (44.2)	2,298,412 (44.2)	1,167,656 (45.7)

Abbreviations: GED, General Educational Development; MBH, mental and behavioral health; MH, mental health
[1]American Indian or Alaska Native alone, Native Hawaiian or Other Pacific Islander alone, other race alone
[2]This is a composite outcome as described in the methods
[3] Observations with "not insured" for Insurance Coverage status excluded

© 2024 Foster AA et al. *JAMA Netw Open*.
