# Supplementary material for: Comprehensiveness of State Insurance Laws and Perceived Access to Pediatric Mental Health Care
Source: JAMA Netw Open. 2024 Aug 12;7(8):e2426402. doi: 10.1001/jamanetworkopen.2024.26402 (PMC11320173; doi:10.1001/jamanetworkopen.2024.26402)
Supplement: Supplement 2. — Data Sharing Statement [file jamanetwopen-e2426402-s002.pdf]

Data Sharing Statement

Foster. Comprehensiveness of State Insurance Laws and Perceived Access to Pediatric Mental Health Care. *JAMA Netw Open*. Published online August 12, 2024. doi:10.1001/jamanetworkopen.2024.26402

Data

Data available: No

Additional Information

Explanation for why data not available: The State Mental Health Insurance Laws Dataset (SMHILD) is shared with researchers upon request and execution of a data use agreement. Interested parties are provided with the full legal data set, the legal coding protocol, and a codebook. The SMHILD lead investigator (MD) is available for technical assistance and consultation upon request. The National Survey of Children's Health is a publicly available dataset (https://mchb.hrsa.gov/data/national-surveys).
